# Pelvic Inflammation in the Female

**Published:** 1893-07

**Authors:** W. C. Fisher

**Affiliations:** Galveston


For Texas Medical Journal.

### PELVIC INFLAMMATION IN THE FEMALE.

BY W. C. FISHER, M. D.

[Read at Meeting of the Galveston County Medical Society.]

THE organs and tissues that form the internal pelvis are so intimately related anatomically, that it has occurred to me to treat them as a whole. This applies particularly to the organs of generation; what affects one of these, in a great measure, either directly or indirectly, affects the other. Most of my remarks, however, will be confined to the tissues that cover and bind together these organs. There are those who classify pelvic inflammation as cellulitis, peritonitis, metro-peritonitis, etc., and doubtless there are cases where this is correct; but it is often the case where it is hard to say where cellulitis merges into peritonitis, and *vice versa.* I think in all probability, nearly all our cases of so-called cellulitis and peritonitis have their origin in some septic or traumatic influence in the uterus and tubes, and by the absorbents this sepsis is carried to the peritoneum that covers the parts, and to the tissue that connects them; therefore, believing they all originate from the same causes, and because they are so closely related anatomically, I think it best to treat them as one, and not endeavor to differentiate them.

The most prolific source of serious pelvic disease, is gonorrhœa, first attacking the vagina, then the mucous membrane of uterus, on to the mucous membrane of the tubes, there producing a virulent form of inflammation, which affects all the surrounding parts. It has been said that gonorrhœa causes more deaths by those inflamed conditions which it leaves in its track, than does its dread confrere, syphilis. This I believe to be undoubtedly true of gonorrhœal inflammation in the female; a large proportion of tubal and ovarian desease of such a serious nature as to require laparotomy, are the result of this poison, hence it behooves us to treat with much care cases of vulval and vaginal gonorrhœa, in the hope that we may be able to check its destructive march before it invades the limited walls of the uterus and tubes. I also believe that many of our cases of endometritis, were the truth known, are so obstinate and so incurable, or so prone to re-invasion, because the gonorrhœal virus still holds on with stubborn grasp. Another prolific source is, lack of cleanliness and proper observation of asepsis in cases of abortion, either as the result of ignorance, laziness or carelessness, or stubbornness on the part of some "old fogy," who has treated a large number of cases in a period of twenty-five or thirty years, with a written report of many recoveries and an unmentioned and unwritten report of a large number who never lived to report the cause of their death.

Individuals of this kind always remind me of the old saying, "convince a woman against her will, and she's of the same opinion still."

The growing desire to be fashionable, to keep up with the demands of society, to limit the household to husband and wife, with perhaps a concession of one, or at most two, children, has rendered the modern society woman the possessor of a conscience so elastic that the murder of the innocent and unborn infant has no horror for her. She will resort to the most nauseous and poisonous draughts to try to dislodge this innocent being from his natural resting place. She will use all contrivances, from a knitting needle to a pen-holder, thrust rudely against the uterus or into the cervical canal. All these failing, as a dernier resort she consults and places herself in the murderous hands of the lowest and most depraved being,—a disgrace to mankind and an unfit associate for the lowest animal of God's creation,—the abortionist. All these means and appliances are used with utter disregard to cleanliness, asepsis, and with no regard to the anatomy of the parts, hence they produce, either from filth, traumatism, or neglect to remove the secundines, a violent septic inflammation, which, in all probability, will either result in death or chronic invalidism. The physician is generally consulted in these cases, after the trouble has reached its acme, for the reason that they desire to hide their crime, or attribute their condition to natural consequences, and not to the true condition of affairs. There are many other causes, but space prevents our further ventilation of causes.

Unfortunately, we meet these inflammatory conditions of the pelvis in their chronic stage, or after the inflammation has reached its height, where there is either a large effusion of lymph, or a still further process, the formation of pus. If we are so fortunate as to recognize a case in the first, or congestive stage, by absolute rest, both physical and physiological, attention to the general condition, and the plentiful irrigation of the parts, we may possibly abort the inflammation, and our patient have no ill effects. But if it goes on to the stage of lymph formation, the same line of treatment holds, but unfortunately it is seldom that these patients make an absolute recovery, either due to non-absorption of this effusion, which presses the surrounding organs out of their proper position, or causing much pain and discomfort, or by bands of adhesion, which prevent the normal movements, which often cause so much pain and agony as to demand grave surgical procedure.

We now come to the last stage, that of suppuration, which in its deadly march sends many to the "happy hunting ground," makes many the victims of a horrible existence, with all the trials and tribulations consequent on sepsis, unless a kind and considerate providence directs its path outward through one or more of nature's channels, or unless the deft hand of the surgeon gives a free outlet, or unless dame nature encysts this pus in a sac with thick walls and poor absorbative powers. We must not be too precipitate in our desire to find concealed pus. Unless we can locate it positively, or unless our patient is in immediate danger from sepsis, we are not justified in opening the abdomen. When there is decided pointing or fluctuation in the vagina, or anywhere else, we are not only justified in making a free opening, but if we fail in so doing, we are acting in direct oppositon to the dictates of our surgical conscience. Before closing this paper, I will mention that it has been my lot, in the past three years, to meet three cases of pelvic suppuration, two of these cases occurring within a month of each other, where the pus was discharged plentifully through the bladder with the urine, with wonderful relief to the individuals. No. 1 being a case resulting from some septic influence in the hands of the midwife in attendance, patient making, after the rupture, a complete recovery, and has given birth to one or two children since. No. 2 gave a history, following confinement, of numerous pelvic abscesses, opening through the walls of the vagina, and afterwards, when under my observation, the discharge as stated before. This individual died several weeks ago, I understand, of uræmic coma, and this was no surprise to me, for she was also a victim of chronic nephritis. No. 3, history of pelvic trouble following confinement about six weeks. She had well-marked symptoms of sepsis, and marked physical signs. After rupture, disappearance (gradual) of septic symptoms, and rapid improvement, on a much reduced economy, and I have reason to suspect that she is now pregnant two months.

These cases have suggested to me that it is one of the best avenues of escape, and unless there be a cystitis, or some break in the continuity of the mucous membrane of the bladder, I do not believe it will become infected.

Another case, illustrating the occasional lack of symptoms of a serious nature, occurred in my practice some months since. I was called to O. R., about twenty years, and found her suffering with a mild form of pelvic inflammation. After a few days of rest, she apparently made a good recovery. From four to six weeks thereafter, I was called to see the same individual, and found her with a well defined tumor, located in the right ovarian region, which felt very much like a simple ovarian cyst. She had no temperature, very little pain, and no symptoms whatever of sepsis. After consultation, I decided to perform laparotomy, which I did, and found a sac containing at least one pint of offensive pus (the sac rupturing and discharging into the cavity). I removed the same rapidly, irrigated with large quantities of boiled water, and my patient made an uninterrupted recovery.

Thanking you, gentlemen, for your forbearance, I leave the subject for your discussion.

